# Heatstroke-induced acute kidney injury and the innate immune system

**DOI:** 10.3389/fmed.2023.1250457

**Published:** 2023-08-08

**Authors:** Hiroyasu Goto, Manabu Kinoshita, Naoki Oshima

**Affiliations:** ^1^Department of Nephrology and Endocrinology, National Defense Medical College, Tokorozawa, Japan; ^2^Department of Immunology and Microbiology, National Defense Medical College, Tokorozawa, Japan

**Keywords:** heat-related illness (HRI), macrophage, neutrophil, NKT (natural killer T) cells, mast cell (MC), NK cells

## Abstract

Heatstroke can cause multiple organ failure and systemic inflammatory response syndrome as the body temperature rises beyond the body’s ability to regulate temperature in a hot environment. Previous studies have indicated that heatstroke-induced acute kidney injury (AKI) can lead to chronic kidney disease. Therefore, there is an urgent need to elucidate the mechanism of heatstroke-induced AKI and to establish methods for its prevention and treatment. Recent reports have revealed that innate immunity, including neutrophils, macrophages, lymphocytes, and mast cells, is deeply involved in heat-induced AKI. In this review, we will discuss the roles of each immune cell in heat-induced renal injury and their potential therapeutic use.

## Introduction

Due to global warming and heat waves, humans are being increasingly exposed to high temperatures, and the rate of heat-related illnesses is consequently increasing as well.

Heatstroke (HS), which is the most severe condition of heat-related illness, is characterized by central nervous system dysfunction and a core body temperature of greater than 40°C (1). Normally, the body temperature is maintained at approximately 37°C. However, when heat gain exceeds heat loss (noncompensatory phase), the body temperature continues to rise, triggering circulatory disturbances, cytotoxicity, and maladaptive inflammatory responses, which induce multiorgan dysfunction, including acute kidney injury (AKI) (2). Recent studies have shown that HS-induced AKI is not temporary but actually progresses to chronic kidney disease (CKD) (3, 4). Therefore, there is an urgent need to elucidate the exact mechanisms underlying HS-induced AKI and to explore prevention and treatment strategies for HS-induced AKI.

Many studies have reported that HS can cause a systemic inflammatory response syndrome (SIRS), which is defined as ≥2 of the following: body temperature >38°C or <36°C, heart rate >90/min, respiratory rate >20/min or PaCO_2_ <32 mmHg, and white blood cell count >12,000/mm^3^ or <4,000/mm^3^ or >10% immature bands (5). SIRS can cause severe shock, disseminated intravascular coagulation (DIC), multiple organ dysfunction, and death (6). Proinflammatory cytokines, such as interleukin (IL)-6, increase following endotoxemia or hyperthermia due to heat exposure (7). Sustained elevation of circulating IL-6 could induce further inflammation and result in poor outcome of HS (8).

Recently, increasing evidence has supported that kidney immune cells play important roles in early tissue injury, repair, and fibrosis after AKI (9). In this review, we will discuss the roles of each immune cell in heat-induced renal injury and therapeutic strategies targeting immune cells.

## Neutrophils

Neutrophils rapidly respond to injured organs. In animal experiments, neutrophils infiltrated injured kidneys as early as 4 h and reached a maximum at 24 h after ischemia-reperfusion injury (10, 11). Consistently, infiltrated neutrophils in the kidneys were also reported to start increasing immediately after heat exposure and increase statistically significantly at 24 h after heat stress (12). It has also been reported that neutrophils increase in bronchoalveolar lavage fluid (BALF) as early as 2 h after heat exposure in a mouse heat-induced acute lung injury model (13). In humans, renal biopsies are rarely performed to confirm the diagnosis of HS-induced AKI, and whether or not neutrophils are involved in AKI after heat stress is unclear. However, one study indirectly showed the relationship between neutrophils and HS-induced AKI. A retrospective study enrolled 187 intensive-care unit (ICU) patients with exertional HS (EHS) and showed that an increase in neutrophils and a decrease in lymphocytes are risk factors for AKI (14).

Neutrophil infiltration into damaged kidneys could contribute to cytotoxicity through phagocytosis, chemotaxis, and oxidative burst (10). A recent study showed that citrullinated histone H3 (Cit-H3), which is involved in the formation of neutrophil extracellular traps (NETs), appeared in the peripheral blood of HS patients (15). In a murine septic AKI model, NETs were induced by necrotic tubule-derived DAMPs and histones, leading to further cytokine release and resulting in additional tubular necrosis (16–18). Furthermore, these Cit-H3-positive cells possessed a multisegmented nucleus, and most were immunoreactive for CD66b (15). Multisegmented neutrophils, the so-called “botryoid nucleus,” were also observed in HS patients and mouse HS models (19, 20). These changes seem to result from nuclear degeneration induced by hyperthermia.

As a therapeutic target, oxytocin has been reported to reduce neutrophil infiltration, myeloperoxidase activity, and oxidative damage markers in acute lung injury after HS (21). Furthermore, CRRT in an early phase (≤8 h after admission) reduced percentages of neutrophils and APACHE II scores, which resulted in an improved survival of HS patients (22).

## Monocytes and macrophages

Mononuclear phagocytes, including monocytes and macrophages, have crucial and distinct roles in tissue homeostasis (23). In experimental animals, several studies have shown that macrophage infiltration into the kidney increased as early as 24 h after heat stress (12, 24). Furthermore, these increases in infiltrated macrophages were associated with the extent of core temperature elevation and exacerbation of tubular damage and fibrosis after heat stress. Using the mitochondrial coupling agent 2,3-dinitrophenol (DNP) with exposure to heat stress, mice expressed higher core temperature, more tubular damage, and more F4/80-positive macrophages in kidney tissue than those who did not use DNP (24).

Kidney macrophages can be classified into two types: bone marrow (monocyte)-derived macrophages and tissue-resident macrophages. Monocytes and bone marrow-derived macrophages are key players in inflammation and pathogen challenge. These cells produce proinflammatory cytokines, such as tumor necrosis factor-α (TNF-α) (25–27). In contrast, tissue-resident macrophages have important roles in the development and resolution of inflammation as well as tissue repair (23, 28–30). Tissue-resident macrophages were reported to enhance Wingless-type MMTV integration site family (Wnt) signaling after injury, which seemed to advance tissue repair (29). Using flow cytometry, we and others have reported that these two subsets of macrophages can be identified based on the differential expression of F4/80 and CD11b in mice: tissue-resident macrophages express F4/80^high^CD11b^low^, and bone marrow-derived macrophages express F4/80^low^CD11b^high^ (27–31). In our previous study, tissue-resident macrophages expressing F4/80^high^ were decreased after heat stress in a mouse heat stress model. These macrophages seemed to become nonviable after heat stress (31). Furthermore, a decrease in the number of tissue-resident macrophages was coincident with a decrease in the number of PCNA-positive tubular cells, indicating delayed tubular regeneration.

Macrophages are also classified into two phenotypes: pro-inflammatory M1 phenotype and anti-inflammatory M2 phenotype (32). This classification was based on the *in vitro* observation that macrophages activated classically [activated by interferon (IFN)-γ] or alternatively (activated by IL-4/IL-13) (32). We and others have reported that kidney macrophages polarize to the M1 phenotype after heat stress (12, 31). These changes occurred immediately after exposure to heat stress, before monocyte-and bone marrow-derived macrophage infiltration into the kidneys. Furthermore, we also showed that not only bone marrow-derived macrophages but also tissue-resident macrophages were polarized to the M1 phenotype, suggesting that the kidney microenvironment after heat exposure might be involved in these phenotypical changes (31). *In vivo*, however, these phenotypes can coexist, so caution should be practiced when interpreting these results (33). For example, M2 macrophages contribute to both tissue repair and fibrosis in the context of AKI-to-CKD progression (34).

**Figure 1:**
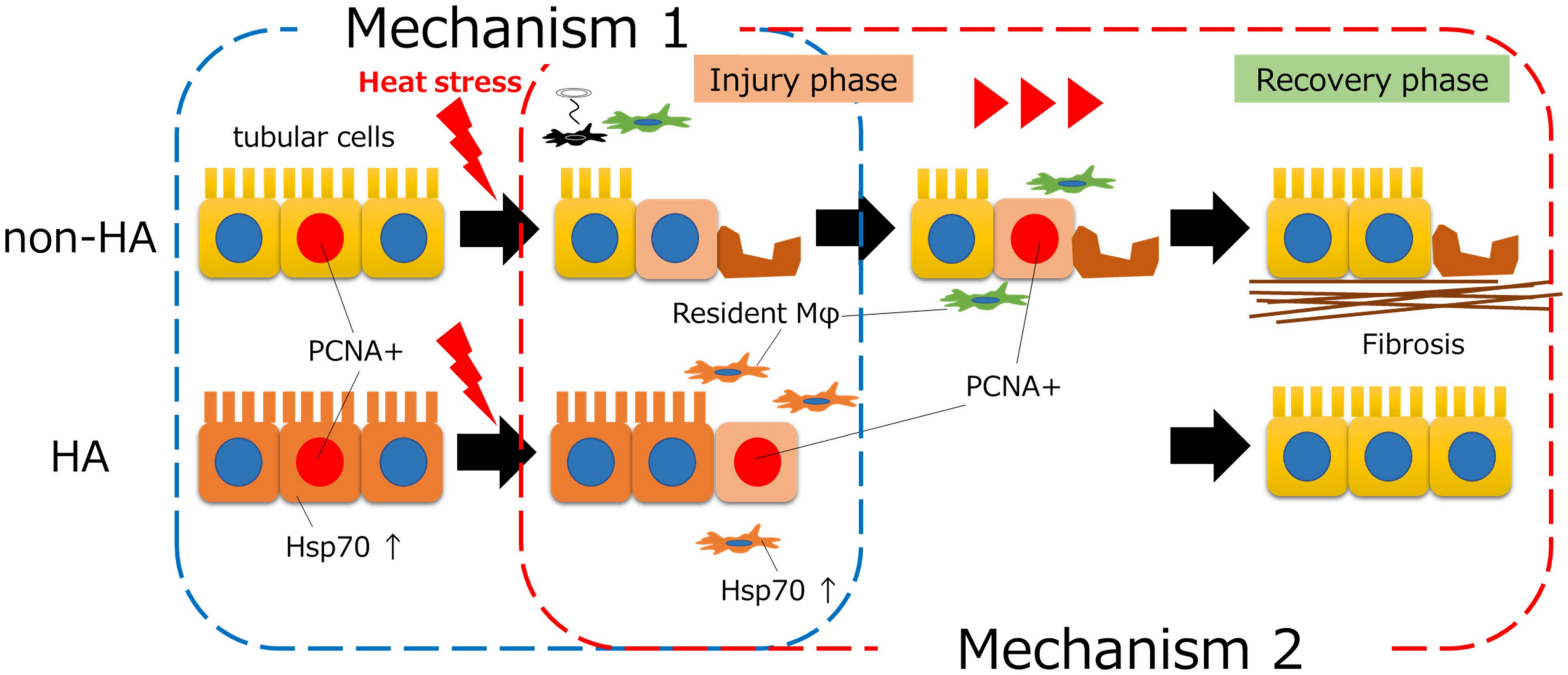
The mechanisms by which heat acclimation (HA) prevents heat stress-induced AKI. HA induced intracellular heat shock protein (Hsp) 70 in tubular cells, which resulted in increased heat tolerance of tubular cells and prevented tubular injury (mechanism 1). HA also increased the levels of intracellular Hsp70 in tissue-resident macrophages and preserved the number of these cells after heat stress. As tissue-resident macrophages play an important role in tissue repair, regeneration of tubules occurred earlier with HA than without HA (mechanism 2).

Regarding therapeutic targets, one study showed that electrical vagus nerve stimulation reduced the infiltration of CD11b-positive macrophages into the lung and spleen, resulting in a decrease in serum proinflammatory cytokines and an improved survival rate in a rat heat stress model (35). We recently evaluated the effect of heat acclimation in mice and found that heat acclimation ameliorated the decrease in tissue-resident macrophages and polarization to the M1 phenotype after heat stress in both bone marrow-derived and tissue-resident macrophages, which improved tubular damage and kidney fibrosis due to heat stress (31). We also found increased levels of heat shock protein (Hsp) 70, which induces stress resilience in each cell, in renal tubular cells and tissue-resident macrophages after heat acclimation. Therefore, heat acclimation can induce cytoprotective effects in tissue-resident macrophages, resulting in preservation of the number of these cells after heat exposure (Figure 1). Furthermore, these results from our study were consistent with a recent clinical study that showed that heat acclimation was associated with a decrease in AKI incidence. The incidence rate of AKI was reported to significantly decrease after a 23 days heat acclimation program in the United Kingdom military, which was coincident with the decrease in the levels of IL-6, one of the proinflammatory cytokines released by M1 macrophages (36).

## Lymphocytes

Lymphocytes also play important roles in HS. Early studies demonstrated that CD8-positive cytotoxic T cells and natural killer (NK) cells are increased in the peripheral blood of HS patients, whereas CD4-positive helper T cells are decreased (37, 38). Furthermore, the number of CD8-positive T cells in the peripheral blood of HS patients was positively correlated with the rectal temperature (37). However, a recent study showed that a decreased number of lymphocytes was associated with increased 90 days mortality (39) and incidence of AKI (15) in HS patients. These controversial results might be due to the focus on whole lymphocytes or specific subpopulations thereof.

NK cells and NKT cells have been reported to play key roles in the early innate response in a mouse AKI model (11, 40, 41). These cells exert cytotoxic activities by releasing cytotoxic mediators and proinflammatory cytokines (42). Furthermore, when NK cells were depleted, the cytotoxic function of NKT cells was enhanced in a mouse α-GalCer-induced AKI model (43).

Regulatory T cells (Tregs), expressing forkhead/winged-helix transcription factor (Foxp) 3, release inhibitory cytokines and negative regulators of inflammation (44). In an animal experiment, decreases in the numbers of splenic Tregs and helper T cells and the production levels of anti-inflammatory cytokines were observed after heat stress (12, 45). Intestinal barrier dysfunction, which can cause leaky gut and endotoxemia, is also affected by a decrease in Tregs after heat exposure (46). These studies implied that Tregs might serve as potential therapeutic targets for HS patients.

## Mast cells

Mast cells play an important role in the innate immune system. They are activated by antigen-specific immunoglobulin (Ig) E via high-affinity receptors for IgE (FcεRI), leading to degranulation and release of mediators, including histamine (47). Furthermore, mast cell-derived cytokines and other mediators affect immune cells, such as dendritic cells, T cells, and B cells (48). A recent study showed that mast cell tryptase (MCT), which is relatively specific for histamine release, was significantly increased in participants who developed post-exercise hypotension compared with those who did not (49). The authors suggest that mast cell degranulation is a vasodilatory mechanism underlying post-exercise hypotension and exercise associated collapse. However, one study showed that heat stress suppressed the IgE-induced degranulation of mast cells (50). A more recent study showed that exercise-associated changes in skeletal muscle temperature generated elevations in intramuscular histamine concentrations, although heating to comparable temperatures did not activate mast cell degranulation in an *in vitro* experiment (51). Therefore, the release of histamine without degranulation may cause post-exercise hypotension. In contrast, some studies have shown that the elevation of histamine levels in skeletal muscle might be important in generating positive adaptations to exercise training (51–53). An increase in histamine concentration induces an increase in post-exercise muscle perfusion (51, 52). Furthermore, with chronic interval training, histamine H1/H2 receptor signaling increases in the skeletal muscle, which results in an enhanced insulin sensitivity, aerobic capacity, and vascular function (NO production) (53). Further studies are needed to investigate whether or not increased histamine and its receptor signaling after chronic training and/or heat acclimation affects systemic adaptation to heat stress.

## Conclusion

It is now clear that HS-induced AKI is due not only to dehydration or a decrease in renal blood flow but also to tubular damage caused by heat stress itself and the inflammatory immune response. Although the contributions of these studies are mentioned above, the precise immune response involved in HS-induced AKI remains unclear due to the wide distribution of involved immune cells and the complex interaction of these cells. Further studies focused on comprehensive changes in kidney immune cells after heat exposure, including studies using single-cell RNA sequencing, are needed to better understand the roles of each immune cell and the interactions of these cells, which might result in the development of new therapeutic approaches.

## Author contributions

HG elaborated this mini-review. HG, MK, and NO edited and revised manuscript. All authors contributed to the article and approved the submitted version.

## Funding

This work was supported by grants from the National Defense Medical College to our department.

## Conflict of interest

The authors declare that the research was conducted in the absence of any commercial or financial relationships that could be construed as a potential conflict of interest.

## Publisher’s note

All claims expressed in this article are solely those of the authors and do not necessarily represent those of their affiliated organizations, or those of the publisher, the editors and the reviewers. Any product that may be evaluated in this article, or claim that may be made by its manufacturer, is not guaranteed or endorsed by the publisher.
